# HOXA4 protein levels and localization in the aorta and in human abdominal aortic aneurysms

**DOI:** 10.1186/1472-6793-11-18

**Published:** 2011-12-14

**Authors:** Christian Klausen, Nelly Auersperg

**Affiliations:** 1 Department of Obstetrics & Gynaecology, Child & Family Research Institute, University of British Columbia, Vancouver, British Columbia, Canada

## Abstract

This report presents evidence for the specificities of select commercially available HOXA4 antibodies in regards to concerns about the specificity of the HOXA4 antibody used by Lillvis *et al. *(**Regional expression of *HOXA4 *along the aorta and its potential role in human abdominal aortic aneurysms**. *BMC Physiol *2011, **11**:9). Using an antibody characterized extensively by us, Lillvis *et al. *report detecting HOXA4 at a size of 33 kDa despite our previous reports that HOXA4 is detected at ~37-39 kDa and that the ~30-33 kDa band is non-specific. Using small interfering RNA targeting HOXA4, forced expression of full-length HOXA4 and HOXA4-positive and -negative ovarian cancer cell lines, we confirm our previous findings that the ~30-33 kDa band is non-specific and that HOXA4 is detected at ~37-39 kDa. Moreover, we demonstrate that HOXA4 small interfering RNA reduces the ~37-39 kDa HOXA4 band, but not the ~30-33 kDa non-specific band, in a human acute monocytic leukemia cell line used by Lillvis *et al. *Western blot analysis performed with two additional commercially available HOXA4 antibodies also detected HOXA4 at ~37-39 kDa. Lastly, immunofluorescent staining of a HOXA4-negative ovarian cancer cell line with the antibody used by Lillvis *et al. *yields strong perinuclear staining, similar to that observed by Lillvis *et al*., which cannot be attributed to HOXA4. Our results highlight and briefly discuss the importance of careful antibody validation and selection for use in various applications.

## Correspondence

We read with interest the study of Lillvis *et al. *[1] regarding the expression of HOXA4 in the aorta and its potential role in abdominal aortic aneurysms. The authors used microarray analysis validated by reverse transcription quantitative real-time PCR to provide strong evidence that HOXA4 mRNA levels are reduced in human abdominal aortic aneurysms relative to control human abdominal aorta. However, we have significant concerns about the subsequent data regarding HOXA4 protein levels. For their studies Lillvis *et al. *used a commercially available rabbit polyclonal HOXA4 antibody (ab26097; Abcam, Cambridge, MA) that was previously characterized extensively by us [2,3]. While they were kind enough to reference our studies, they state that HOXA4 was detected as a single band at ~33 kDa and evidence for this is presented in their Additional file three, Figure S1A. However, in both of our previous studies we state that the size of HOXA4 is ~37-39 kDa and our first study [2] demonstrated that the band at ~30-33 kDa is a non-specific band.

**Figure 1:**
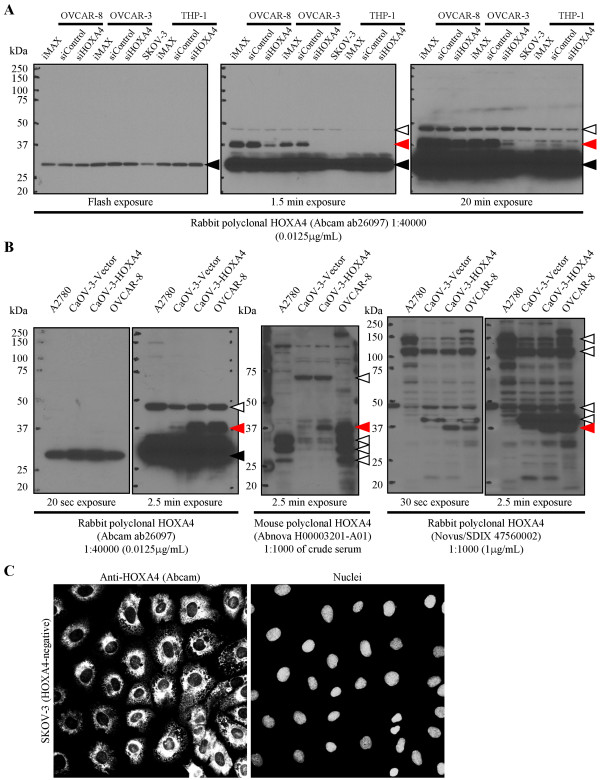
**Evidence for the specificities of select commercially available HOXA4 antibodies**. Immunoblot analysis was performed as in [3] with lysates from one human acute monocytic leukemia (THP-1) and five ovarian cancer (OVCAR-8, OVCAR-3, SKOV-3, A2780 and CaOV-3) cell lines. Red arrowheads indicate HOXA4 (~37-39 kDa), black arrowheads indicate the ~30-33 kDa non-specific band associated with the Abcam antibody, and white arrowheads indicate select non-specific bands of equal/greater intensity. **(A) **SKOV-3 cells lack HOXA4 whereas OVCAR-8 and OVCAR-3 cells express high levels of HOXA4 [2,3]. Transient knockdown was performed as in [3] with 20 nM HOXA4-targeting siRNA (siHOXA4; ON-TARGET*plus *SMARTpool; Dharmacon), control siRNA (siControl; ON-TARGET*plus *Non-Targeting Pool) or transfection reagent alone (iMAX; Lipofectamine RNAiMAX). Immunoblot analysis with the Abcam HOXA4 antibody characterized by us [2,3] shows that the ~30-33 kDa non-specific band detected by Lillvis *et al. *[1] is insensitive to HOXA4 siRNA and is expressed by HOXA4-negative SKOV-3 cells. **(B) **A2780 cells lack HOXA4 whereas CaOV-3 cells express low levels of HOXA4 [2,3]. CaOV-3 cells were transfected as in [3] with control vector (CaOV-3-Vector) or vector encoding full-length HOXA4 (CaOV-3-HOXA4). Immunoblot analysis with the Abcam antibody and two other commercially available HOXA4 antibodies shows that the ~37-39 kDa HOXA4 band detects exogenously expressed HOXA4 and is undetectable in HOXA4-negative A2780 cells. **(C) **Formaldehyde-fixed SKOV-3 cells were permeabilized, blocked and incubated overnight with anti-HOXA4 (1:3200). Secondary antibody was applied, cell nuclei were stained with Hoechst 33258, and coverslips were examined by epifluorescence microscopy. Although HOXA4 mRNA [2,3] and protein (A) are undetectable in SKOV-3 cells, these cells have strong expression of the ~30-33 kDa non-specific band ((A) and [2]). Note that immunofluorescent staining of HOXA4-negative SKOV-3 cells with the Abcam antibody yields strong perinuclear staining which cannot be attributed to HOXA4.

The ~30-33 kDa non-specific band is by far the most intense band and appears as a single band at low exposures irrespective of blotting conditions, as was observed by Lillvis *et al. *in Additional file three, Figure S1A. This was demonstrated previously by us [2] and we now provide additional evidence of this in Figures 1A and 1B. Strong expression of the ~30-33 kDa non-specific band is observed in the five human ovarian cancer cell lines shown in Figures 1A and 1B regardless of the fact that HOXA4 mRNA is undetectable in both SKOV-3 and A2780 cells [2,3]. More importantly, the ~30-33 kDa non-specific band is insensitive to small interfering RNA (siRNA) targeting HOXA4 ([2] and Figure 1A) and forced expression of full-length HOXA4 (Figure 1B). Likewise, Figure 1A shows strong, siRNA-insensitive expression of the ~30-33 kDa non-specific band in the acute monocytic leukemia cell line used by Lillvis *et al. *(THP-1 cells, designated as MP1 cells by Lillvis *et al. *in Additional file four, Table S3).

In contrast, we have demonstrated that the ~37-39 kDa HOXA4 band correlates with HOXA4 mRNA levels, is sensitive to HOXA4 siRNA and detects exogenously expressed HOXA4 ([2,3] and Figures 1A and 1B). Although the ~37-39 kDa HOXA4 band is expressed at very low levels in THP-1 cells, it is the only band that is reduced by treatment with HOXA4 siRNA (Figure 1A). Furthermore, we now provide evidence that two additional commercially available HOXA4 antibodies detect a ~37-39 kDa band that correlates with HOXA4 mRNA levels and detects exogenously expressed HOXA4 (Figure 1B). In this context, we are not convinced that the Western blot results presented by Lillvis *et al. *reflect changes in HOXA4 protein levels. In light of their convincing mRNA data, perhaps a re-examination of the results of longer exposures would allow Lillvis *et al. *to quantify the ~37-39 kDa HOXA4 band detected with this antibody. We routinely cut away the membrane just below the 37 kDa molecular mass marker prior to immunoblotting in order to prevent strong signal from the ~30-33 kDa non-specific band from interfering with quantitation of the ~37-39 kDa HOXA4 band.

Our second, and very much related, concern has to do with the use of the same rabbit polyclonal HOXA4 antibody for immunohistochemistry and immunofluorescence. In light of the intense ~30-33 kDa non-specific band and additional ~46-48 kDa non-specific bands of equal or greater intensity than HOXA4, we have significant concerns about the use of this antibody in these types of applications. Indeed, we have performed immunofluorescent staining of HOXA4-negative SKOV-3 cells ([2,3] and Figure 1A) and found strong perinuclear staining similar to that observed by Lillvis *et al. *which cannot be attributed to HOXA4 since SKOV-3 cells don't express HOXA4 (Figure 1C). While this staining most likely corresponds to the ~30-33 kDa non-specific band due to its intensity and the high dilution of the antibody (1:3200 *vs*. 1:200 used by Lillvis *et al*.), we cannot rule out contributions from the ~46-48 kDa non-specific bands. Nuclear localization, especially as assessed by epifluorescence rather than confocal microscopy, does not appear to be sufficient to identify HOXA4 in cells with demonstrated HOXA4 expression since Lillvis *et al. *also report detecting the ~30-33 kDa band in both cytoplasmic and nuclear fractions by Western blot.

In this context, we are not convinced that the immunohistochemistry and immunofluorescence results presented by Lillvis *et al. *accurately depict the localization of HOXA4 protein. Indeed, we would suggest that the use of this rabbit polyclonal HOXA4 antibody in these types of applications is unwarranted unless definitive validation of specificity is presented. Our preliminary Western blot analysis of two additional commercially available HOXA4 antibodies has similarly revealed a number of non-specific bands of equal or greater intensity than HOXA4 (Figure 1B). Persuasive validation studies will therefore be required to establish whether any of the commercially available HOXA4 antibodies possess the specificity required for use in applications such as immunohistochemistry and immunofluorescence. Future studies on the biology of HOXA4 and its roles in disease would benefit greatly from the development of a highly specific antibody suitable for use in these and other applications.

The example of HOXA4 highlights the need for researchers to validate the specificity of antibodies for use in various applications, especially in the absence of rigorous antibody characterization. Indeed, while the Abcam HOXA4 antibody performs very well in Western blot analysis, its use in other applications such as immunocytochemistry, immunohistochemistry or immunoprecipitation is likely unwarranted unless definitive validation studies are performed in consideration of the sheer intensity of the signal from the ~30-33 kDa protein. For many companies antibody characterization takes the form of ELISA or Western blot analysis with the antigenic peptide/protein. While this does establish whether or not the antibody can detect the particular peptide/protein against which it was raised, it does not prove it will detect the actual protein (except when raised and tested against full-length protein) nor does it address how the antibody will perform in the context of the entire proteome (i.e. in cells or tissues) or in different applications. Preabsorption studies with the antigenic peptide/protein can identify *true *non-specific bands (i.e. cannot be eliminated by saturation), but they cannot distinguish the protein of interest from non-specific proteins with similar epitopes that the antibody binds to in a specific manner. For example, preabsorption of the Abcam HOXA4 antibody with the antigenic peptide completely eliminates the ~37-39 kDa HOXA4 band, the ~30-33 kDa non-specific band and one of the ~46-48 kDa non-specific bands [2]. Thus, the other ~46-48 kDa non-specific band is likely a *true *non-specific band, yet this approach fails to identify only the ~37-39 kDa band as being specific for HOXA4.

The detection of a band of "appropriate" size in cell or tissue lysates can be used to confirm the identity of a band, but must involve more than one (preferably three or more) lysates from cells or tissues *known *to have differing basal or experimentally manipulated expression levels. In general, accurate up-front knowledge of expression levels is most easily achieved by measuring mRNA levels. An obvious, and highly effective, extension of this approach is to use RNA interference-mediated knockdown or vector-based forced expression to generate lysates with gene-specific changes in the expression level of the protein of interest. Although use of an antibody in several applications requires separate validation for each application, many of the approaches discussed above can be adapted to different applications. In addition, companies are beginning to employ these approaches as they strive to market gene-specific product portfolios (antibody, protein/peptide, RNA interference and forced expression) on a genome-wide scale. However, in the absence of thorough antibody characterization it is ultimately the responsibility of the researcher to ensure the validity of results obtained from antibody-based applications.

## Competing interests

The authors declare that they have no competing interests.

## Authors' contributions

CK conceived of the study, participated in its design, carried out all experiments and drafted the manuscript. NA participated in the design of the study and helped to draft the manuscript. All authors read and approved the final manuscript.
